# ASSOCIATION BETWEEN SCHOOL DESEGREGATION AND THE COGNITION OF OLDER BLACK MEN: A CITY-LEVEL ANALYSIS

**DOI:** 10.1093/geroni/igad104.3192

**Published:** 2023-12-21

**Authors:** Collin Perryman, Taharka Anderson, Roland J Thorpe, Keith Whitfield

**Affiliations:** Johns Hopkins Bloomberg School of Public Health, Baltimore, Maryland, United States; University of Texas at Austin, Austin, Texas, United States; Johns Hopkins University, Baltimore, Maryland, United States; University of Nevada, Las Vegas, Las Vegas, Nevada, United States

## Abstract

**Objective:**

We sought to understand the relationship between school setting attendance (desegregated or segregated) and the cognition among older Black men.

**Methods:**

Data from older Black men (n=153; age range: 50 to 89 years) from the Baltimore Study of Black Aging—Patterns of Cognitive Aging (BSBA-PCA) was used to explore the relationship between type of schooling and cognitive functioning. We conducted ANOVA and OLS regression analyses to find associations between schooling and various cognitive domains. The independent variable, school type, was measured using a variable assessing if participants attended a racially-mixed school in their youth. The outcome variable, cognition, was measured using global cognition, reasoning, memory, immediate recall, working memory, language, and perceptual speed.

**Results:**

ANOVA analyses found that Black men who attended desegregated schools reported significantly better reasoning and global cognition. OLS regression analyses found that global cognition, reasoning, language, and working memory were significantly better performance in the OLS regression results. The multivariate analysis found that global cognition and reasoning yielded statistically better performance.

**Conclusion:**

Our study provides insights to how cognition varies in Black men depending on the school settings in which they were educated. Additionally, our study finds evidence that past educational policies represent risk for poor cognitive health of older Black men populations.

